# Constitutional mislocalization of Pten drives precocious maturation in oligodendrocytes and aberrant myelination in model of autism spectrum disorder

**DOI:** 10.1038/s41398-018-0364-7

**Published:** 2019-01-17

**Authors:** Hyunpil Lee, Stetson Thacker, Nicholas Sarn, Ranjan Dutta, Charis Eng

**Affiliations:** 1 Genomic Medicine Institute, Lerner Research Institute, Cleveland Clinic, Cleveland, USA; 2 Cleveland Clinic Lerner College of Medicine, Cleveland, OH 44195 USA; 3 Department of Genetics and Genome Sciences, Case Western Reserve University School of Medicine, Cleveland, USA; 4 Department of Neurosciences, Lerner Research Institute, Cleveland Clinic, Cleveland, USA; 5 Germline High Risk Cancer Focus Group, Comprehensive Cancer Center, Case Western Reserve University School of Medicine, Cleveland, USA

## Abstract

There is a strong genetic association between germline *PTEN* mutation and autism spectrum disorder (ASD), making *Pten*-mutant models exemplary for the study of ASD pathophysiology. We developed the *Pten*^*m3m4*^ mouse, where Pten is largely restricted from the nucleus, which recapitulates patient-like, autism-related phenotypes: behavioral changes, macrocephaly, and white matter abnormalities. This study aimed to investigate the contribution of oligodendrocyte (OL) lineage differentiation and functional changes in myelination to the white matter phenotype. OL lineage differentiation and myelination in *Pten*^*m3m4*^ mice was studied using immunohistochemical and electron microscopic analyses. We also used primary oligodendrocyte progenitor cells (OPCs) to determine the effect of the *Pten*^*m3m4*^ mutation on OPC proliferation, migration and maturation. Finally, we assessed the myelinating competency of mutant OLs via co-culture with wildtype dorsal root ganglia (DRG) neurons. The in vivo analyses of *Pten*^*m3m4/m3m4*^ murine brains showed deficits in proteolipid protein (Plp) trafficking in myelinating OLs. Despite the increased expression of myelin proteins in the brain, myelin deposition was observed to be abnormal, often occurring adjacent to, rather than around axons. Mutant primary OPCs showed enhanced proliferation and migration. Furthermore, mutant OPCs matured precociously, exhibiting aberrant myelination in vitro. Mutant OPCs, when co-cultured with wildtype DRG neurons, showed an inability to properly ensheath axons. Our findings provide evidence that the *Pten*^*m3m4*^ mutation disrupts the differentiation and myelination programs of developing OLs. OL dysfunction in the *Pten*^*m3m4*^ model explains the leukodystrophy phenotype, a feature commonly associated with autism, and highlights the growing importance of glial dysfunction in autism pathogenesis.

## Introduction

Autism spectrum disorder (ASD) is a neurodevelopmental disorder characterized by impaired reciprocal social interaction accompanied by restricted interests and repetitive behaviors^1^. As with all complex diseases, there are variable genetic and environmental contributions, however, it is well-established that there is a significant genetic component to ASD. Although the genetic architecture of ASD is complex, there are cases of strong, monogenic associations, such as with *PTEN*. Of children diagnosed with ASD and macrocephaly, 7–17% harbor germline *PTEN* mutations^2–5^. Studying monogenic, syndromic models of ASD may help illuminate shared features of the disorder. Consequently, the constitutional *Pten*^*m3m4*^ model, which recapitulates many of the behavioral, morphological, and molecular features of ASD, has been leveraged to study common mechanisms of ASD pathogenesis^6,7^. Importantly, the neural transcriptome of this mouse reveals differentially expressed genes in common with many known human ASD-related genes^8^.

The *Pten*^*m3m4*^ mouse is a constitutive knock-in model which restricts Pten predominantly to the cytoplasm^6^. White matter abnormalities, one of the hallmarks of ASD, have also been described in patients with germline *PTEN* mutations, as well as the *Pten*^*m3m4*^ model^6,8^. Increased white matter volume is more marked in patients with germline *PTEN* mutations and ASD (PTEN-ASD) than in macrocephalic ASD patients without *PTEN* mutations^8^. The *Pten*^*m3m4*^ mouse has increased proliferation of NG2 glia, increased numbers of oligodendrocyte (OL) lineage cells, significant upregulation of genes involved in central nervous system myelination (accession number GO:0022010), and increased thickness of the corpus callosum without changes in cortical thickness^6,8^. These changes are consistent with an increased white matter volume, but the cellular mechanisms responsible require elucidation.

The advantage of using the *Pten*^*m3m4*^ model to study OL development and function is that the *Pten*^*m3m4*^ model is a germline knock-in mutation that closely mimics the molecular and neurological phenotypes of patients with PTEN-ASD. Our central hypothesis is that germline *Pten*^*m3m4*^ mutation affects OL development and subsequent OL dysfunction contributes to the ASD phenotype by not only disrupting myelination, but also by altering neuronal physiology, such as axon pathfinding. Here, we show through in vivo and in vitro studies that the constitutional disruption of Pten nuclear localization results in dysregulated development and function of OLs.

## Materials and methods

See the Supplemental Information for the complete details of the techniques outlined below.

### Animals and reagents

Generation and characterization of the *Pten*^*m3m4*^ mouse has been described previously^6^. All experiments were conducted under protocols approved by the Institutional Animal Care and Use Committee (IACUC) at Cleveland Clinic. Mice were maintained on a 14:10 light: dark cycle with access to food and water ad libitum. The room temperature (RT) was maintained between 18 and 26 °C. Animals were euthanized via CO_2_ asphyxiation or exsanguination via transcardial perfusion with phosphate-buffered saline (PBS). For the histological and electron microscopy, we used only male mice. While performing in vitro experiments, we observed the same phenotypes for both sexes across all experiments, but greater variation in the white matter phenotype among females. Hence, we used both female and male mice but conservatively utilized more female samples than male for the primary OPC culture-related experiments (F > M).

### Immunohistochemistry (IHC)

Immunohistochemical analysis was performed as previously described^6^. Brains were transcardially perfused with phosphate-buffered saline (PBS) and fixed with 4% formaldehyde for overnight. Brains were post-fixed in the same fixative for 24 h, and then dehydrated in 30% sucrose before sectioning on a cryostat. All sections were 10 μm coronal sections cut using a Leica VT1200 S Vibratome (Leica Biosystems, Buffalo Grove, IL).

### Immunofluorescence staining

Immunofluorescence labeling was performed by incubating tissue sections with primary antibody and then with fluorochrome-conjugated secondary antibody. The sections were mounted using VECTASHIELD Mounting Medium with DAPI (Vector Laboratories) for fluorescence applications. Images were analyzed using a Leica Laser Confocal Microscope (Leica Biosystems, Richmond, IL).

### Electron microscopy

Mice were perfused with cold PBS followed by modified Karnovsky’s fixative (2% paraformaldehyde/2.5% glutaraldehyde). The brain was removed and post-fixed overnight in the same fixative at 4 °C. Corpus callosum was isolated from 1 mm coronal sections of brain between −0.94 and −2.18 of bregma.

### Purification and culture of OPCs

OPCs were separated from the primary mixed glial cell cultures of cerebral hemispheres of 2-day-old mouse pups as previous described^9^. OL cultures were typically >95% pure as assessed by immunocytochemistry for the OL lineage marker NG2 and the astrocyte marker glial fibrillary acid protein (GFAP).

### Cell counting

To quantify the total density of OLs and OPCs in control mice relative to mutant mice, we performed whole slide scanning and counted NG2-positive and CC1-positive cells in cerebral cortex, corpus callosum, and hippocampal parenchyma in micrographs taken with a ×5 objective (five or six fields in five and six sections from each of two mice). The numbers were normalized and are quoted in the results as Olig2-positive cells per mm^3^.

### Determination of cell proliferation by Ethynyl-2′-Deoxyuridine (EdU) Incorporation

To label the proliferating OPCs, 10 μM EdU was added to the culture medium for 20 h at 37 °C and washed twice in 1× PBS. Finally, the cells were fixed in ice-cold 100% methanol for 10 min at −20 °C and EdU-labeled cells were detected according to the manufacturer’s instructions of the Click-iT EdU Imaging Kit (Invitrogen, Waltham, Massachusetts, USA).

### Differentiation assay

Differentiation of OPCs was induced by the addition of thyroid hormone^10^ (T3 at 30 ng/ml) and PDGF withdrawal^11,12^. Differentiated OLs were counted, based on their characteristic morphology^12,13^.

### OPC migration assay

The Boyden chamber migration assay was used to determine direct migration of OPCs. Isolated OPCs were seeded in the upper compartment of the Boyden chamber. Only the medium in the bottom well contained the PDGF and bFGF, which were used as OPC chemoattractants^14,15^, thus, allowing the OPCs to migrate to the lower compartment containing PDGF and bFGF (10 ng/ml) for 24 h at 37 °C in 5% CO_2_.

### Oligodendrocyte and dorsal root ganglion neuron co-culture in vitro

Dorsal root ganglion (DRG) neurons were isolated from 2 week-old murine spinal cord regions as previously described^16^ and grown in dispersed cultures on PDL-coated coverslips for 10 days to establish dense beds of axons. OPCs from *Pten*^*wt/wt*^, *Pten*^*wt/m3m4*^, and *Pten*^*m3m4/m3m4*^ mice were seeded onto neurons and grown for 7 days.

### Western blot analysis

Cerebral cortex was lysed in RIPA buffer (20 mm Tris-HCl, pH 7.5, 150 mm NaCl, 1 mm EDTA, 1 mm EGTA, 1% NP-40, 1% DOC) supplemented with protease inhibitor (Sigma) and phosphatase inhibitor 2, 3 (Sigma). Samples were analyzed with a standard Western blot protocol.

## Results

### *Pten*^*m3m4*^ mutation alters oligodendrocyte lineage development

**Fig. 1:**
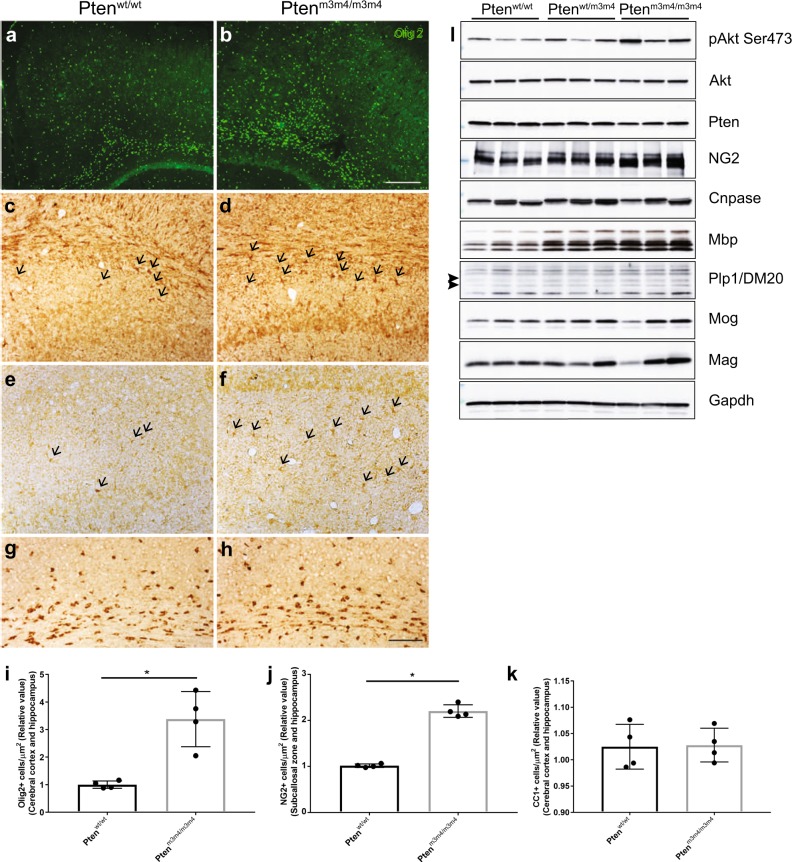
Increased oligodendrocyte progenitor cells (OPCs) without increased mature oligodendrocytes (OLs) in *Pten*^*m3m4/m3m4*^ mice. Olig2+ cells in the cerebral cortex and corpus callosum of (**a**) *Pten*^*wt/wt*^ and (**b**) *Pten*^*m3m4/m3m4*^ mice. NG2+ cells in the subcallosal zone (SCZ) of (**c**) wildtype and (**d**) homozygous mutant mice. NG2+ cells in the hippocampal zone (HZ) of (**e**) wildtype and (**f**) homozygous mutant mice. Mature oligodendrocytes (OL) labeled CC1+ cells in deep cortex and corpus callosum of (**g**) wildtype and (**h**) *Pten*^*m3m4/m3m4*^ mice. IHC results were quantified for (**i**) Olig2+ in the cortex, (**j**) NG2+ in SCZ and HZ, and (**k**) CC1+ cells in the cortex. (**l**) Western blot analysis on cerebral cortex from P14 *Pten*^*m3m4/m3m4*^ mice for P-Akt Ser473, total Akt, Pten, NG2 (OPC marker), Cnpase (immature OL marker), and major myelin proteins (Plp, Mbp, Mog, and Mag). Results represent mean ± SD; *p < 0.05; Mann–Whitney Test

Our previous studies using the *Pten*^*m3m4*^ mice have shown increased brain size and proliferation of the OL lineage cells^6^. Studies of ASD patients with germline *PTEN* mutations have reported white matter abnormalities^8^. This increased proliferation with white matter abnormalities, led us to investigate OL lineage in detail. We performed immunohistochemistry (IHC) with Olig2, in the brains of two-week-old *Pten*^*m3m4*^ mice. Significant increases of Olig2 expression were found in the cerebral cortex including the corpus callosum of *Pten*^*m3m4/m3m4*^ mice compared to that of wildtype age-matched littermates (Fig. 1a, b, i). Our results provide evidence of increased OL lineage cells in the OPCs of *Pten*^*m3m4/m3m4*^ mice. We next investigated whether this increased OPC population also leads to increased maturation to OLs using NG2 and CC1 antibodies, which are markers for OPCs and mature OLs, respectively. NG2-positive cells were mainly detected in the subcallosal zone (SCZ) of the wildtype mouse brain. However, the number of NG2-positive cells was increased not only in SCZ (Fig. 1c, d, j), but also in the hippocampal parenchyma of *Pten*^*m3m4/m3m4*^ mice (Fig. 1e, f, j). No differences were observed in CC1-positive cells across the different mouse genotypes (Fig. 1g, h, k). Taken together, the increase in Olig2 and NG2 indicate increased OPC proliferation in *Pten*^*m3m4/m3m4*^ mice without concomitant maturation and increase in the number of OLs.

We further validated the increase in NG2 levels using Western analysis (Fig. 1l). This increase in NG2 was also accompanied by an increase in the phosphorylation of Akt at Ser473 in cortex of *Pten*^*m3m4/m3m4*^ mouse brain and decreased Pten levels (Fig. 1l and Supplementary Fig. S1a–c), a result previously demonstrated in the original characterizations of the model^6^. Investigating further, the level of Cnp expression, an immature OL marker, however was found to be unchanged (Fig. 1l and Supplementary Fig. S1d) in the *Pten*^*m3m4/m3m4*^ mouse cortex. We next investigated the levels of myelinating OL markers: myelin basic protein (Mbp), proteolipid protein (Plp), myelin oligodendrocyte protein (Mog) and myelin associated glycoprotein (Mag) in the cortex of the *Pten*^*m3m4/m3m4*^ mice. By visual inspection all these markers increased in the mutant brains, and all of these markers except Mog showed a significant increase in the *Pten*^*m3m4/m3m4*^ mouse brain compared to that of wildtype mice (Fig. 1l and Supplementary Fig. S1e–h). We detected a significant increase in both isoforms of Plp, the longer isoform called Plp1 and the shorter isoform DM20, though there was no significant change in the Plp1/DM20 ratio across genotypes (Fig. 1l and Supplementary Fig. S1h–j). The increase in myelin proteins continued as six-week old mice also showed a striking increase in Mbp and Plp in the cortex of the homozygous mutant (Supplemental Fig. 2). Taken together these results therefore provide evidence of increased proliferation of OPCs with increased myelin protein synthesis without concurrent increase in the number of mature OLs.

In order to explain the observation of increased OPC proliferation without change in mature OLs, we explored OL apoptosis in *Pten*^*m3m4*^ mice using anti-cleaved caspase-3 and Plp antibodies. We found increased co-localization of cleaved caspase-3 with Plp, with more cleaved caspase-3 staining in the brains of heterozygous and homozygous mutants compared to wildtype littermates (Supplementary Fig. S3a). Western analysis demonstrated increased cleaved caspase-3 expression in the brain with increasing dosage of the m3m4 allele (Supplementary Fig. S3b, c). These results provide evidence that OLs undergo apoptosis in Pten^m3m4^ mice, and this may explain the lack of an increase in OL numbers.

### Abnormal morphology of myelinating oligodendrocytes in *Pten*^*m3m4/m3m4*^ mice

**Fig. 2:**
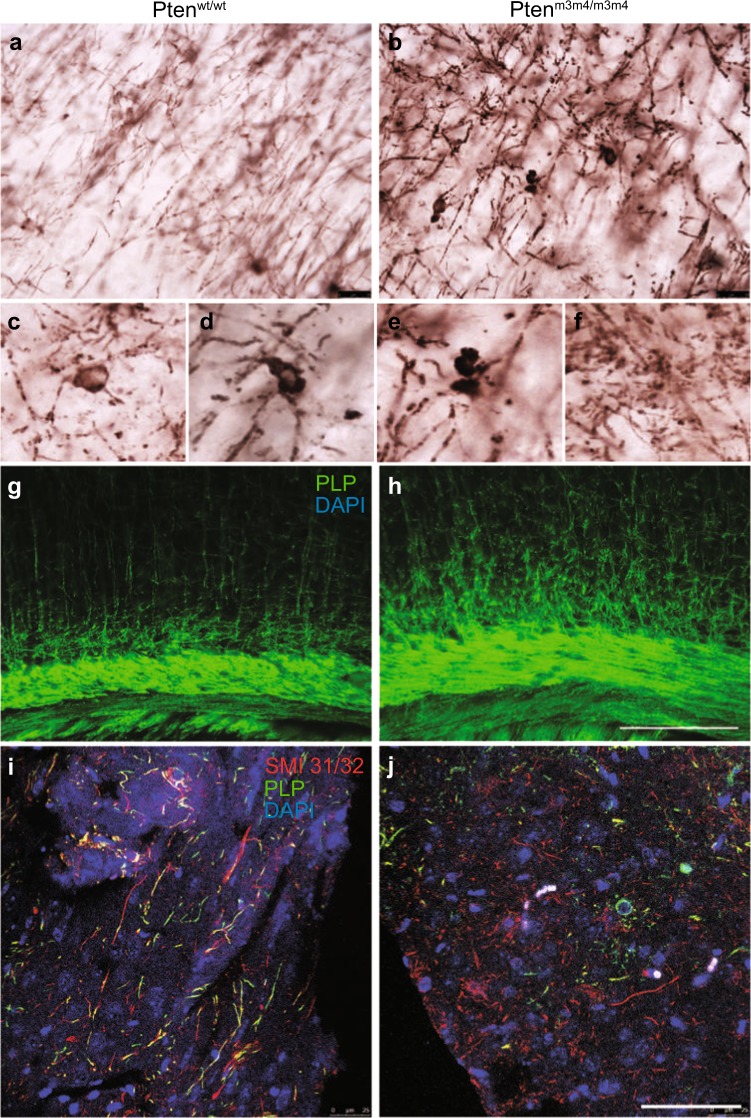
Abnormal morphology of myelinating oligodendrocytes (OLs) in *Pten*^*m3m4/m3m4*^ mice. High-magnification images of Plp+ myelinating OLs in cortex of (**a**) P14 wildtype and (**b**) age-matched *Pten*^*m3m4/m3m4*^ mice showing Plp+ myelin on axons in wildtype animals. (**c**), (**d**), (**e**), and (**f**) are high magnification images from (**b**). Immunofluorescence staining of Plp in (**g**) wildtype and (**h**) *Pten*^*m3m4/m3m4*^ mice reveals Plp staining on branched and complexed morphology of axons in homozygous mutant mice. Immunofluorescence co-staining of SMI 31/32 and Plp on the brains of (**i**) wildtype and (**j**) *Pten*^*m3m4/m3m4*^ mice reveal Plp not co-localized with SMI 31/32 in *Pten*^*m3m4/m3m4*^ mice in comparison to wildtype mice. Scale bar = 25 μm (**a, b**); 20 μm (**g, h**); 50 μm (**i, j**)

Increased expression of myelin proteins without an increase in CC1-positive mature OLs in *Pten*^*m3m4/m3m4*^ mice directed our focus to myelinating OLs. Because Plp is the major protein in myelinating OLs, we performed IHC for Plp in two-week old *Pten*^*m3m4*^ mice. In the wildtype mouse brain, Plp staining was detected evenly along axons (Fig. 2a) compared to the homozygous mutant mouse brain where the staining was detected within the intracellular clump, condensed cell body, or fragmented processes of myelinating OLs (Fig. 2b, c–f). From the Plp immunofluorescence staining experiment, we observed a more complex branching pattern of myelin on axons in the homozygous mutant compared to wildtype (Fig. 2g, h). To examine myelination of axons in *Pten*^*m3m4*^ mice, we co-stained for axons and myelin using the axonal marker SMI31/32 and the myelin marker Plp. The results show complete co-localization of SMI 31/32 with Plp in wildtype mice; however, in the mutant mouse, we detected almost no co-localization between the myelin (Plp) and axons (SMI31/32) (Fig. 2i, j). These results provide evidence to support the hypothesis that there are deficits in the myelination program of homozygous mutant OLs despite their increased production of myelin proteins.

### Aberrant myelination in central nervous system white matter in *Pten*^*m3m4*^ mice

**Fig. 3:**
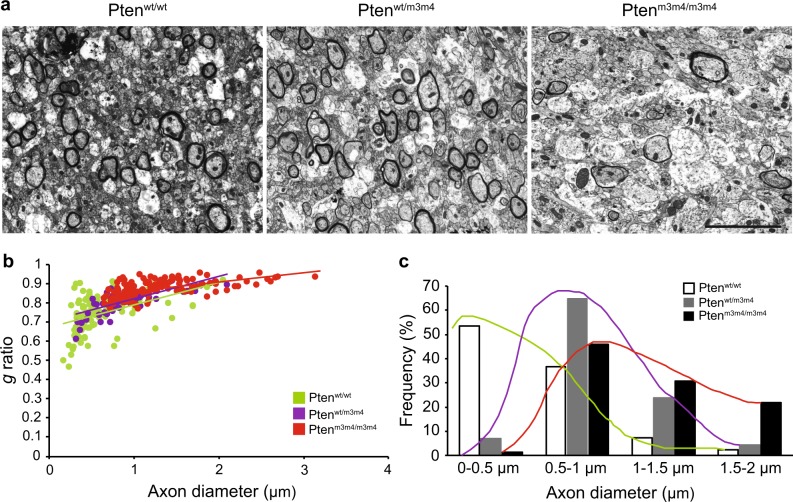
Dysmyelination of the corpus callosum in *Pten*^*m3m4/m3m4*^ mice. **a** Electron micrographs of corpus callosum in sagittal section of two-week-old *Pten*^*wt/wt*^, *Pten*^*wt/m3m4*^ and *Pten*^*m3m4/m3m4*^ mice (*n* = 3 per genotype) revealed the evident thinning of myelin sheaths in *Pten*^*m3m4/m3m4*^ mice. **b** Scatter diagram shows the distribution of the *g* ratio as a function of axon diameter in each of the genotypes. The *g* ratio and axon diameters were plotted for individual axons. **c** Quantification of the number of axons in different size distribution for each genotypes. Scale bar = 5 μm (**a**)

To further investigate myelin integrity in the *Pten*^*m3m4*^ mutant mice, we performed electron microscopy (EM) on the corpus callosum of two-week-old mice. Interestingly, the EM data revealed decreased myelin sheath thickness and an increased *g*-ratio with increasing dosage of the m3m4 allele (Fig. 3a, b). The increased *g*-ratio is explained by the simultaneous decrease in ensheathed myelin and increase in axonal caliber (swelling). As axonal caliber was significantly increased in *Pten*^*m3m4*^ mice, we quantified axonal diameter in the mutant mice. The results show that mutant mice have an abundance of large diameter axons compared to those of wildtype mice (Fig. 3c). Collectively the results provide evidence that successive reduction in nuclear Pten leads to successive increase in axonal diameter. This change in axonal caliber is accompanied by attenuated myelination of axons despite increase myelin protein expression.

### *Pten*^*m3m4*^ mutation enhances oligodendrocyte precursor cell proliferation and migration in brains

**Fig. 4:**
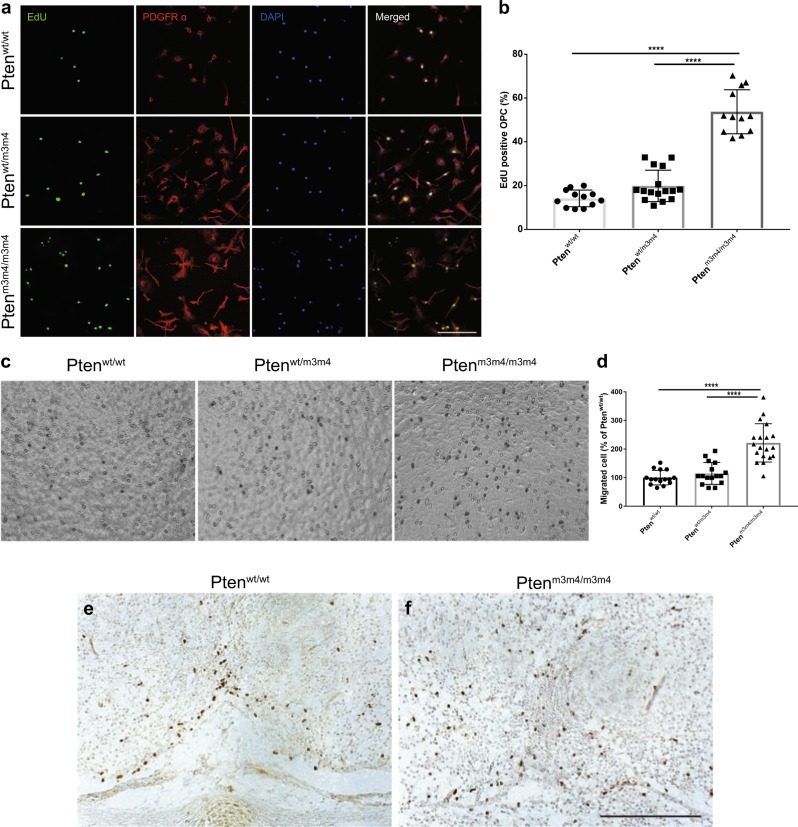
Increased oligodendrocyte progenitor cell (OPC) proliferation and migration in *Pten*^*m3m4/m3m4*^ mice. **a** Representative images of EdU incorporation assay of OPCs isolated from *Pten*^*wt/wt*^*, Pten*^*wt/m3m4*^, and *Pten*^*m3m4/m3m4*^ mice. **b** Quantification of the EdU-positive cells. EdU-positive cells were counted from 12 to 16 different fields in each of the 3 genotypes under 20x magnification. OPCs were isolated from 3 or 4 different animals in each group with mixed sex. **c** Photomicrograph of cultured OPCs that have transmigrated through the transwell membrane. **d** Quantification of transmigrated OPCs from each genotypes revealed that OPC from *Pten*^*m3m4/m3m4*^ mice revealed increased migration compared to those of *Pten*^*wt/wt*^. Images of Olig2 IHC in the embryonic diencephalon (E14.5) of (**e**) wildtype and (**f**) *Pten*^*m3m4/m3m4*^ demonstrated that OPC proliferation and migration are abnormally regulated in the embryonic diencephalon from the *Pten*^*m3m4*^ homozygous mutant mice. Scale bar = 100 μm (**a**). Scale bar = 200 μm (**e, f**). ****p < 0.0001; One-way ANOVA

The OPC phenotypes in the *Pten*^*m3m4*^ model suggest defects not only in lineage development, but also in proliferation and migration. We, therefore, isolated OPCs from both wildtype and *Pten*^*m3m4*^ mice at postnatal day 2 (P2) to characterize their proliferation and migration. To do this, we employed an Edu labeling assay and observed that the number of EdU-positive cells was significantly increased in mutant OPC cultures compared to wildtype OPC cultures (Fig. 4a, b). Subsequently, we performed migration assays using a Boyden chamber and observed an increased number of transmigrated cells in homozygous mutant OPCs compared to heterozygous mutant or wildtype OPCs (Fig. 4c, d). The in vitro proliferation and migration data are consistent with our in vivo data on migration. At E14.5, we found an irregular distribution OPCs throughout the diencephalon in addition to greater OPC number. Therefore, we posit that the cellular programs controlling OPC proliferation and migration are impaired in *Pten*^*m3m4*^ mice (Fig. 4e, f).

### *Pten*^*m3m4*^ mutation leads to precocious maturation but disrupts myelin membrane spreading during oligodendrocyte maturation

**Fig. 5:**
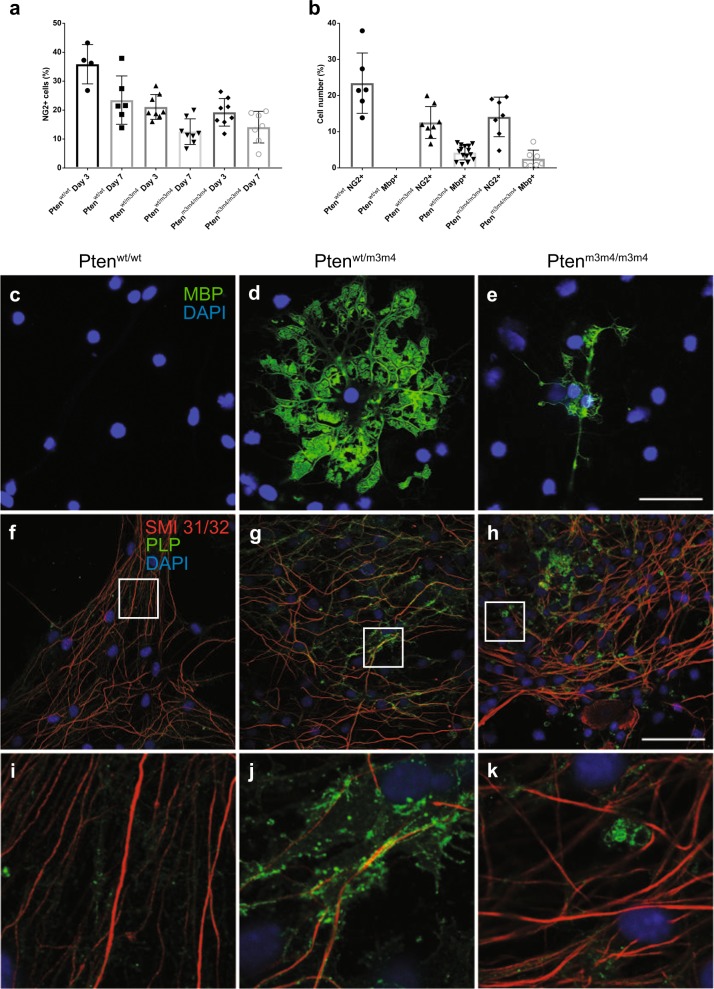
Precocious maturation and impaired myelination in *Pten*^*m3m4/m3m4*^ mice. **a** NG2+ cells were counted at 3 and 7 days of differentiation. As differentiation goes on, the number of NG2-positive cells was decreased which was further decreased in *Pten*^*wt/m3m4*^ and *Pten*^*m3m4/m3m4*^ compared to those of wildtype. **b** Quantification of NG2+ and Mbp+ cells counted at 7 days of differentiation. At 7 days, we could not detect any Mbp+ cells in wildtype cultures. However, we found that approximately 5% and 3% of cells were Mbp+ in heterozygous and homozygous mutant OLs at 7 days of differentiation, respectively. **c–e** Representative images showing myelin spreading in Mbp+ mature OLs at 7 days of differentiation across model genotypes. Wildtype DRG neurons at DIV 10 were co-cultured with (**f**) wildtype, (**g**) heterogyzous mutant, and (**h**) homozygous mutant OLs for 7 days. (**i**), (**j**), and (**k**) are high magnification images boxed in panel (**f**), (**g**), and (**h**), respectively. Representative images show that SMI-31/32 (red), axonal maker and Plp (green), for marking myelin membranes. Scale bar = 50 μm (**c–k**)

Next, we evaluated differentiation by culturing OPCs and probing for NG2 and Mbp. NG2-positive cells were gradually decreased as differentiation progressed (Fig. 5a). There were no Mbp-positive cells at 3 days of differentiation. However, we found that approximately 5% and 3% of cells were Mbp-positive in heterozygous and homozygous mutants at 7 days of differentiation, respectively (Fig. 5b). At 7 days, we could not detect any Mbp-positive cells in wildtype cultures. The early Mbp expression indicates accelerated maturation in the mutant cultures. Interestingly, myelin membrane spreading in *Pten*^*wt/m3m4*^ OLs is precocious and aggressive, whereas myelin spreading in *Pten*^*m3m4/m3m4*^ is precocious but dysfunctional (Fig. 5c–e). Therefore, these data suggest that the maturation process is accelerated in the mutant OPCs but is modulated by mutation dosage.

In order to determine the myelinating capacity of mature OLs, we co-cultured mutant OPCs with DRG neurons isolated from wildtype mice. As such, Plp-positive myelin membranes were not obvious on DRG neurons cultured with wildtype OLs at 7 days of co-culture (Fig. 5f, i); however, intact and aggressive Plp-positive myelin membranes were found along axons cultured with *Pten*^*wt/m3m4*^ heterozygous OLs (Fig. 5g, j). The Plp-positive myelin membranes from homozygous mutant OLs were not ensheathing axons as much as heterozygous mutant OLs (Fig. 5h, k). This is reflected in the quantified myelination index, indicating a lower score in the homozygous mutant (Supplementary Fig. S4). In the homozygous mutant co-culture, it appears as if the myelin is deposited adjacent to but not wrapped around the axons. These data demonstrate that oligodendrocytes from the *Pten*^*m3m4*^ homozygous mutant do not properly target nor subsequently ensheath axons with myelin, an observation consistent with our EM data (Fig. 3).

## Discussion

This study demonstrates that constitutional mutation of *Pten* resulting in intracellular mislocalization dramatically changes OL lineage progression, morphology, and myelination. First, we found evidence for an increase in OL lineage cells in the *Pten*^*m3m4/m3m4*^ mouse, specifically an increase in proliferation of OPCs without an increase in OLs (Fig. 1). This finding was partially explained by the increased apoptosis that was observed in OLs in the *Pten*^*m3m4/m3m4*^ brain (Supplementary Fig. S3). In addition to the changes in OL lineage, we found a marked increase in the expression of myelin proteins in the *Pten*^*m3m4/m3m4*^ brain (Fig. 1 and Supplemental Figs. 1, 2). Furthermore, we found that myelinating OLs in homozygous mutant mice display abnormal morphology; the myelin is improperly deposited in the cell body of OLs, failing to ensheath axons (Fig. 2). In investigating the dysmyelination phenotype further, we found increases in axonal caliber without concomitant increases in myelin sheath thickness (Fig. 3). In vitro, we found increased proliferation and migration in mutant OPCs (Fig. 4a–d). We also observed that mutant OPCs mature precociously and exhibit increased myelin production that is accompanied by deficits in myelin spreading (Fig. 5a–e). Finally, we observed that culturing mutant OLs with wildtype DRG neurons captured the inability of the *Pten*^*m3m4/m3m4*^ OLs to properly myelinated axons. It appears that axon pathfinding may be disturbed in the DRG neurons when cultured with the mutant OLs (Fig. 5f–k). Ultimately, these findings illustrate the multitudinous contributions of Pten to OL development and function, specifically the myelination program, which appears to be deeply dependent on robustly functioning Pten.

This study illustrates the unique effects that constitutional Pten mislocalization has on the myelination program of OLs. We chose to study myelination at two weeks of age in the *Pten*^*m3m4*^ model because this roughly corresponds to the age of PTEN-ASD patients, who are overwhelmingly in the pediatric age group, and it is often difficult to obtain desired sample sizes at six weeks of age due to the premature mortality observed in *Pten*^*m3m4*^ mice. In addition, the white matter abnormalities observed at six-weeks of age, exemplified, in part, by over-expression of myelin proteins (i.e., Plp1 and Mbp), are consistent with those observed at the two-week timepoint (Supplementary Fig. S2). While the current study provides insight at a snapshot in time, and we believe a critical time, it does have limitations in providing detailed insight into how the myelination program may change as the *Pten*^*m3m4*^ mouse ages in the context of affecting behavioral outcomes in a longitudinal manner.

The critical role of Pten in myelination has been previously demonstrated by the germline model of constitutively active Akt and various conditional knockout models of Pten in OLs. These models show hypermyelination with increased myelin protein expression^17–21^. *Pten*^*m3m4*^ mice also display increased white matter volume and elevated levels of myelin proteins (Fig. 1 and Supplemental Fig. S1, S2); however, the *Pten*^*m3m4*^ model does not simply recapitulate a hypermyelination phenotype. The mutant OLs appear to have difficulties with myelin-spreading consistent with the observed aberrant morphology, the clumping of Plp adjacent to, but not, wrapped around axons. This dysmyelination implicates Pten in myelin-spreading mechanisms, such as the trafficking of myelin proteins. Our study does not interrogate the role of Pten in mechanisms of myelin-spreading, but exploring the role of Pten in intracellular trafficking may be fruitful given the known role of phosphoinositides in directing intracellular cargo^22^. The pathophysiological effects of the dysmyelination require more examination, but the inability to properly myelinate axons appears to be leading to apoptosis in mature OLs (Supplementary Fig. S3). Furthermore, the myelination phenotype is exaggerated in the in vitro data relative to our in vivo data, and these data insinuate that the mutant OLs may alter axon caliber and pathfinding (Fig. 5). The predominant model of OL and axon interaction places greater regulatory burden on axons. Axons have been shown to regulate OPC proliferation and OL myelination dynamics and survival^23^. However, ablation of OLs in the cerebellum of postnatal rodents altered neuronal circuitry and caused dysregulation of genes related to axonal growth and guidance, thus suggesting a regulatory role for OLs^24^. Moreover, it is well known that after traumatic brain injury, OLs suppress new axonal growth^25^. Thus, the effects of mutant OLs on axonal growth and guidance warrant further study, especially in the context of Pten signaling, which may be therapeutically targetable for axon regeneration.

This study also illustrates the unique effects that the *Pten*^*m3m4*^ mutation has on OL lineage differentiation. Pten conditional knockout models have also described changes in OL lineage differentiation, but the advantage of the germline *Pten*^*m3m4*^ model is that it allows for a comprehensive analysis of OL progression in the constitutional context, similar to the human germline *PTEN* mutation. Thus our findings related to changes in OL lineage differentiation are distinct from those described by the studies on the *Olig2-cre:Pten*^*fl/fl*^ and *Pdgfra-CreER; R26-EYFP; Pten*^*fl/fl*^ models. The *Olig2-cre:Pten*^*fl/fl*^ mouse has increased OPC numbers, but those OPCs do not show an increased capacity for proliferation^19^, speaking to the importance of timing and perhaps of context. In contrast, the *Pdgfra-CreER; R26-EYFP; Pten*^*fl/fl*^ mouse shows increased OPC number and proliferation, but these authors argue that the proliferation of OPCs serves to enhance OPC-to-OL conversion, leading to a proportional increase in OLs^21^. However, we observe no change in OL number in the *Pten*^*m3m4/m3m4*^ brain, though this may be explained by the increased apoptosis (Fig. 1a–k and Supplementary Fig. S3). The disparate observations hint that context, timing, and cell-non-autonomous factors are important, also providing clues to the difference between loss-of-function mutations and *Pten* mutants exhibiting mislocalization. Ultimately, our data suggest the m3m4 mutation affects OL lineage progression in a manner that is subtly distinct from *Pten* knockouts either in the neural stem cell (NSC) pool destined to become OLs (i.e., *Olig2*) or in OPCs (i.e., *Pdgfra*). In contrast to the conditional models, it is difficult to identify whether cell-autonomous mechanisms or external factors are driving the OPC phenotypes observed. Further studies are warranted to elucidate the mechanisms responsible for the subtle differences in OPC proliferation across the different models. The role of PTEN as a sentinel against unchecked proliferation has implications beyond the development of the nervous system, most notably in neoplastic conditions, like Cowden Syndrome^26^. Studies of PTEN and cell proliferation should carefully consider PTEN dosage, which has been correlated with cancer risk in murine models^27^ and with phenotype burden in patients with germline *PTEN* mutations, at least statistically^4^.

In this study, we traced OL lineage development, examined OPC proliferation and migration, and characterized white matter abnormalities by exploring myelination dynamics in *Pten*^*m3m4*^ mice (summarized in Supplementary Fig. S5). We have demonstrated that constitutional cellular mislocalization of Pten disrupts significant aspects of OPC and OL physiology with dramatic effects on myelination, which occurs precociously and presents with aberrant morphology. This OL pathophysiology contributes to the gross white matter abnormalities and likely contributes significantly to the behavioral phenotype of the model. In fact, the white matter phenotype of *Pten*^*m3m4*^ mice, especially the morphology and white matter quality of the corpus callosum, is extremely reminiscent of the white matter phenotype of PTEN-ASD patients^8^. Although this study cannot distinguish between the cell autonomous mechanisms and external factors that may contribute to OL pathophysiology, it is nonetheless important evidence that the mislocalization of Pten can dramatically perturb OL development and myelination, contributing to neuroanatomical abnormalities associating with ASD. These findings may be highly relevant to ASD patients with *PTEN* mutations that display the same mislocalization of PTEN. Furthermore, this study demonstrates the emerging importance of glia to ASD pathogenesis in the context of germline *PTEN* mutation.

## Supplementary information

Supplemental Materials
