# Supplementary material for: Constitutional mislocalization of Pten drives precocious maturation in oligodendrocytes and aberrant myelination in model of autism spectrum disorder
Source: Transl Psychiatry. 2019 Jan 17;9:13. doi: 10.1038/s41398-018-0364-7 (PMC6341090; doi:10.1038/s41398-018-0364-7)
Supplement: Supplementary file 1 — Supplemental Materials [file 41398_2018_364_MOESM1_ESM.docx]

**Supplementary Information**

**Constitutional mislocalization of Pten drives precocious maturation in oligodendrocytes and aberrant myelination in model of autism spectrum disorder**

Hyunpil Lee^1^, Stetson Thacker^1, 5^, Nicholas Sarn^1, 2^, Ranjan Dutta^4, 5^, Charis Eng^1, 2, 3, 5^

**MATERIALS and METHODS**

**Animals and reagents**

The segment of murine *Pten* exon 7 was altered via knock-in, inactivating two of the four nuclear localization sequence-like sequences (NLS) within exon 7 and 8 is required for disrupting proper subcellular localization of PTEN.^1^ Genotypes of all mice were determined by PCR analysis of tail genomic DNA based on the suggested Jackson Laboratory protocol with modified PCR primers. PCR primers specific for wildtype alleles are as follows: mPTEN-F5, 5′-TGGCAGACTCTTCATTTCTGTGGC-3′, and mPTEN-R6, 5′-ACTTCTTCACAACCACTTCTTTCAAC-3′; for mutant alleles, they are as follows: mPTEN-F3, 5′-TACCCGGTAGAATTTCGACGACCT-3′, and mPTEN-R6, 5′-ACTTCTTCACAACCACTTCTTTCAAC-3′.

Recombinant human PDGF-AA, basic FGF, and NGF were purchased from PeproTech (Rocky Hill, NJ, USA). The following antibodies were used: anti-proteolipid protein (PLP)/DM20 antibody (Clone AA3), anti-NG2 antibody (rabbit, Millipore, Cleveland, OH, USA, AB5320), anti-CC1 antibody (Mouse mAb, Millipore, OP80), anti-MBP antibody (Mouse mAb, Santa Cruz Biotech, Santa Cruz, CA, USA, sc-271524), anti-MBP antibody (Rat, Millipore, MAB386), anti-Olig2 antibody (Rabbit pAb, Millipore, AB9610), anti-myelin oligodendrocyte glycoprotein (MOG) antibody (Abcam, Cambridge, MA, USA no. 32760), anti-myelin associated glycoprotein (MAG) antibody (Cell Signaling, Danvers, MA, #8043), anti-phospho-Akt Ser473 (Cell Signaling, #9271), anti-Akt antibodies (Cell Signaling, #4691), anti-phospho-ERK1/2 (Cell Signaling, #9101), anti-ERK1/2 (Cell Signaling, #9102), anti-PTEN antibody (Cascade Biosciences, Winchester, MA, USA, 1:5000), anti-αTubulin (Sigma, St Louis, MO, USA, T6074), and anti-SMI-31/32 (Biolegend, Dedham, MA, USA, #801601/#801701).

**Immunohistochemistry**

Brains were transcardially perfused with phosphate-buffered saline (PBS) and fixed with 4% formaldehyde for overnight. Brains were post-fixed in the same fixative for 24 hours, and then dehydrated in 30% sucrose before sectioning on a cryostat. After removal, the brain was transferred to 10% sucrose and further incubated for 24 hours to cryoprotect. The brain was cut in 10 μm coronal sections. The sections were mounted on glass slides and stored at -80 °C until they were used. Immunohistochemistry (IHC) was performed using the ABC method. Briefly, sections were dehydrated through descending series of ethanol, and finally into Tris buffered saline (TBS: 50 mM Tris, 150 mM NaCl, pH = 7.6). Endogenous peroxidase was removed with a 30-minute incubation in 3% H_2_O_2_. Antigen retrieval using citrate buffer and a pressure cooker (Biocare, Pacheco, CA) was performed for most IHC experiments. After blocking for 30 minutes in 10% normal goat serum (NGS), primary antibodies were applied and incubated overnight at 4 °C. After washing, coverslips were incubated for 60 minutes at room temperature in goat anti-mouse IgG-biotin or goat anti–rabbit IgG-biotin (1:1000). Finally, sections were incubated with a mixture of either FITC-streptavidin or Texas red–streptavidin (both at 1:100) for 30 min at RT. The sections were developed with ABC (Vector Laboratories, Burlingame, CA) and slides were rinsed in dH_2_O and then mounted. For Plp immunohistochemistry, free-floating coronal sections (30 μm) were analyzed, with antigen retrieval in 10 mm sodium citrate (pH 6.0) at 65°C for 10 min as needed, using a Pelco Biowave Pro tissue processor (Ted Pella, Redding, CA, USA). For IHC, OLs were cultured on coverslips (see Primary cell culture and electroporation, below) and fixed with ice-cold methanol for 10 min at -20°C. Cells were permeabilized with 0.3% Triton X-100 for 15 min at RT, blocked with 10% NGS for 60 min at RT, and incubated with primary antibodies overnight at 4°C.

**Immunofluorescence staining**

Immunofluorescence labeling was performed by incubating tissue sections with primary antibody and then with the fluorochrome-conjugated secondary antibody. Alexa fluoro-anti-mouse IgG 488 (FI-1000; Vector Laboratories) or Alexa fluoro-anti-rabbit IgG 568 (Vector Laboratories, TI-2000) were used. For double labeling, the sections were washed with 0.1% Tween in PBS after the first primary antibody, and immunostaining was repeated with a different set of primary and secondary antibodies, as indicated in the figure legends. Negative controls were performed in parallel by omitting the primary antibodies. The sections were mounted using VECTASHIELD Mounting Medium with DAPI (Vector Laboratories) for fluorescence applications. Images were analyzed using a Leica Laser Confocal Microscope (Leica Biosystems, Richmond, IL).

**Electron microscopy**

Mice were perfused with cold PBS followed by modified Karnovsky's fixative (2% paraformaldehyde/2.5% glutaraldehyde). The brain was removed and post-fixed overnight in the same fixative at 4 °C. Corpus callosum was isolated from 1 mm coronal sections of brain between −0.94 and −2.18 of bregma. All tissue was post-fixed in 1% osmium tetroxide, dehydrated in graded acetone, and resin embedded in Embed 812 (Electron Microscopy Sciences) using a Pelco Biowave Pro tissue processor (Ted Pella, Redding, CA, USA). The corpus callosum samples were oriented such that sections could be cut midline in a sagittal plane. Ultrathin sections (80 nm) were mounted on copper grids, stained with uranyl acetate and lead citrate, and viewed at 80 kV on a Tecnai G2 transmission electron microscope (FEI company, Hillsboro, OR). Electron micrographs of the corpus callosum were imaged at the midline.

**Purification and culture of oligodendrocyte progenitor cells (OPCs)**

The primary mixed glial cell cultures were prepared from cerebral hemispheres of 2-day-old mouse pups and cultured for 9 days in T75 flask. On the tenth day, the cell cultures with 10 ml of fresh medium were placed into an incubator and allowed to equilibrate for 2 hours. Once the caps were completely tightened, the flasks were shaken for 40 min at 110 rpm to remove loosely attached microglia and floating cells. The cultures were then replaced with fresh media and followed by shaking for 15-18h at 240 rpm, 37 °C as previous described.^2^ This procedure usually began in the afternoon so that the cultures were shaken overnight. Following the shaking period, the suspended cells from the flasks were collected by centrifuging for 5 min at 1200 rpm. At this point, OL cultures were typically >95% pure as assessed by immunocytochemistry for the OL lineage marker NG2 and the astrocyte marker glial fibrillary acid protein (Gfap). Cells were resuspended and seeded onto poly-D-lysine-coated dishes or round 12 mm coverslips in DMEM F12 supplemented with N2 (Life Technologies, #17502-048), B27 (Life Technologies, #17504-044), Basic fibroblast growth factor (bFGF, 10 ng/ml), Platelet-derived growth factor (PDGF, 10 ng/ml), and penicillin-streptomycin (GIBCO BRL). Cultures were maintained at 37 °C in a 5% CO_2_ incubator, and half of the culture medium was changed every other day. For differentiation, OPCs were incubated in differentiation media in which PDGF, bFGF, and B27 were removed and added T3 (30 ng/ml), CNTF (30 ng/ml) and Insulin (100 ng/ml) 2 days after plating.

**Cell counting**

For quantification of OLs and OPCs, we counted Olig2+ cells in fluorescence microscope images taken with a 20x objective (at least three fields from three or more 20 μm sections taken from one or two mice). For each experiment, the data are presented as the proportion of total Olig2-positive cells that were also NG2-positive or CC1-positive. To quantify the total density of OLs and OPCs in control mice relative to mutant mice, we performed whole slide scanning and counted NG2-positive and CC1-positive cells in cerebral cortex, white matter tract (corpus callosum) and hippocampus in micrographs taken with a 5x objective (five or six fields in five and six sections from each of two mice). The numbers were normalized and are quoted in the results as Olig2-positive cells per mm^3^.

**Determination of cell proliferation by Ethynyl-2′-Deoxyuridine** (**EdU) incorporation**

To label the proliferating OPCs, cells were plated at a density of 3,000 cells/12-mm PDL-coated glass coverslip and cultured for 2 days in medium containing PDGF plus bFGF. Then 10 μM EdU was added to the culture medium for 20 hours at 37 °C and washed twice in 1X PBS. Finally, the cells were fixed in ice-cold 100% methanol for 10 minutes at -20 °C and EdU-labeled hcells were detected according to the manufacturer’s instructions of the Click-iT EdU Imaging Kit (Invitrogen, Waltham, Massachusetts, USA). Ten microscopic fields were visualized from each experimental group with cells and the proportion of PDGFR-positive/EdU-positive cells was expressed as the ratio of total cells counted in each field.

**Differentiation assay**

Differentiation of OPCs was induced by the addition of thyroid hormone^3^ (T3 at 30 ng/ml) and PDGF withdrawal.^4, 5^ In order to quantitate the differentiation response, OPCs (2x10^4^ cells/ well) were plated into PDL-coated glass cover slips and cultured for 2 d in PDGF plus bFGF. Then, culture medium was switched to differentiation media including T3 was added to the culture medium and without PDGF and bFGF. Half of the culture medium was replaced with fresh medium every 3 d. After 3 days in vitro (DIV) and 7 DIV, cells were fixed with ice-cold methanol for further immunocytochemical detection. Differentiated OLs were counted, based on their characteristic morphology.^6. 7^

**OPC migration assay**

The Boyden chamber migration assay was used to determine direct migration of OPC. Isolated OPCs were resuspended in DMEM/F12 and then 5 × 10^4^ cells in 50 μL of DMEM containing 0.5% FBS were seeded in the upper compartment of the Boyden chamber containing an 8 μm diameter polycarbonate membrane with polyvinylpyrrolidone surface treatment. Only the medium in the bottom well contained the PDGF and bFGF, which were used as OPC chemoattractants.^7, 8^ thus, allowing the OPCs to migrate to the lower compartment containing PFGF and bFGF (10 ng/ml) for 24 hours at 37 °C in 5% CO_2_. Membranes were removed from the Boyden chamber, fixed and non-migrated cells from the upper surface of the membrane were discharged by gentle scraping and rinsing. Remaining migratory cells were stained with Giemsa and the number of migrated cells counted.

**Oligodendrocyte and dorsal root ganglion neuron co-culture *in vitro***

Dorsal root ganglion (DRG) neurons were isolated from 2 week-old murine spinal cord regions as previously described^9^ and grown in dispersed cultures on PDL- coated coverslips for 10 days to establish dense beds of axons. OPCs from *Pten^wt/wt^*, *Pten^wt/m3m4^*, and *Pten^m3m4/m3m4^* mice were differentiated for 4 days prior to seeding onto neurons and grown for 7 days. Every other day, half of the media was changed with fresh neurobasal media including NGF (50 ng/ml). Co-cultures were fixed 7 days after OPC addition, stained for PLP (1:200) and SMI 31/32 (1:1000), and nuclei were stained with DAPI. The coverslips were mounted with Vectashield reagent containing 4′,6-diamidino-2-phenylindole (DAPI; Vector Laboratories). The secondary antibodies were as follows: Alexa Fluor 488 goat anti-rat IgG (H+L), and Alexa Fluor 563 goat anti-mouse IgG (H+L). The fluorescence images were captured with Leica confocal microscopy system (DMI4000B; Leica, DeltaVision Elite Imaging System; GE Healthcare) and analyzed with AF6000 software (Leica).

**Western blot analysis**

Cerebral cortex was lysed in RIPA buffer (20 mM Tris-HCl, pH 7.5, 150 mM NaCl, 1 mM EDTA, 1 mM EGTA1% NP-40, 1% DOC) supplemented with protease inhibitor (Sigma) and phosphatase inhibitor 2, 3 (Sigma). Samples were analyzed by a standard Western blot protocol. For Pten pathway analysis, 30 μg of purified protein per sample was run on a 4–12% acrylamide gel (Criterion, Hercules, CA, USA) before being transferred to a nitrocellulose membrane. Membranes were blocked for 1 hour in 5% non-fat milk / 1X TBST before being incubated in primary antibody diluted in 3% BSA overnight at 4°C. The primary antibodies used in this study were mouse anti-human PTEN (Cascade Biosciences, 1:5000), rabbit anti-phosphorylated-ERK1/2 (Cell Signaling, 1:2000), rabbit anti-total ERK1/2 (Cell Signaling, 1:2000), rabbit anti-total Akt (Cell Signaling, 1:2000), rabbit anti-phosphorylated Akt Ser473 (Cell Signaling, 1:250), rabbit anti-myelin associated glycoprotein (MAG; Cell Signaling Technology, #8043), rabbit anti-2′, 3′-cyclic-nucleotide 3′-phosphodiesterase (CNPase; Cell Signaling Technology, #5664), rabbit anti-myelin oligodendrocyte glycoprotein (MOG; Abcam, #32760), mouse anti-MBP (Santa Cruz Biotechnology, #sc-272524), and rabbit anti-GAPDH (Cell Signaling, 1:20,000). Anti-rabbit or mouse IgG conjugated HRP antibodies were used at a concentration of 1:5,000, and membranes were incubated for 1 h at room temperature. The blots were incubated with West Pico ECL solution for 3 min at RT to develop the chemiluminescent signals (Thermo Fisher, Waltham, MA, USA). Membranes were imaged using Amersham Imager 600 and band intensity analysis was using Quantity One (BioRad) software.

**Data analysis**

Data are expressed as the mean ± SD and analyzed using a one-way (analysis of variance (ANOVA), followed by post-hoc Tukey-Kramer analysis or Student’s t-test where appropriate (Graph Pad Prism 7). The non-parametric tests, Mann-Whitney and Kruskall-Wallis test were used where appropriate (Graph Pad Prism 7). P-values less than 0.05 were considered statistically significant.

**SUPPLEMENTARY FIGURES**

**
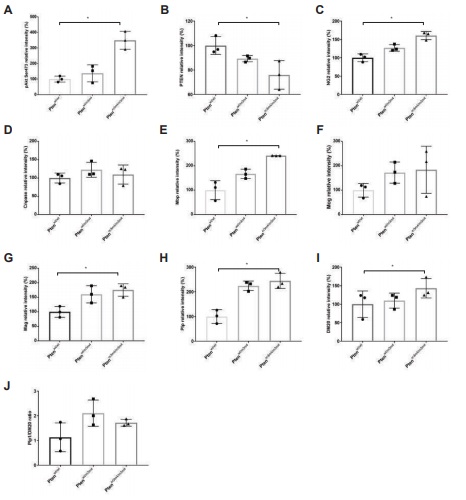
**

**Supplemental Figure S1.** Quantification of western blot on myelin protein expression (Manuscript Figure 1L). Quantification of P-Akt Ser473 (A), Pten (B), NG2 (C), Cnpase (D), Mbp (E), Mog (F), Mag (G), Plp1 (H), DM20 (I) and Plp1/DM20 ratio (J) from Western blot data. Individual intensity of wildtype samples was set to 1 based on averaging intensity of bands and heterozygous and homozygous mutant values were graphed as percentage of control. Bar graphs represent mean ± SD, n=3, * p<0.05; Kruskal-Wallis test.

**
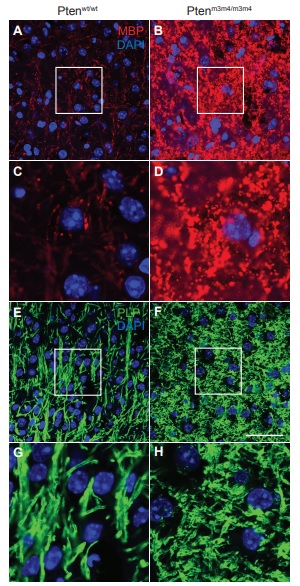
**

**Supplemental Figure S2.** Myelin-related proteins were dysregulated in the cortex of *Pten^m3m4/m3m4^* mice at P40. Mbp-positive immunoreactivities in wildtype (A) and *Pten^m3m4/m3m4^* mice (B) showed significant different patterns between two groups. (C) and (D) panels were magnified from boxed regions of panel (A) and (B), respectively. Plp stained along the axons in wildtype (E). Plp+ axonal morphology was obviously dysregulated in *Pten^m3m4/m3m4^* mice (F). (G) and (H) panels were magnified images from boxed regions of panel (E) and (F), respectively. Scale bar = 50 μm.


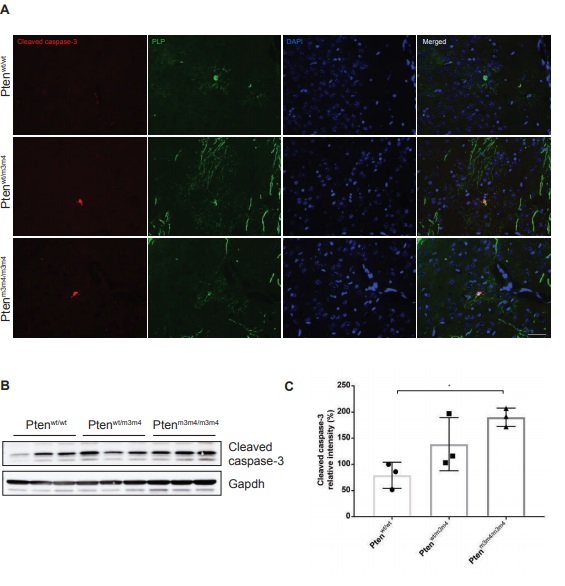


**Supplemental Figure S3.** Loss of mature oligodendrocytes (OLs) in *Pten^m3m4/m3m4^* mice. (A) Images of double staining with cleaved caspase-3 (red) for labeling apoptotic cells and Plp (green) on the brains of two-week-old mice demonstrated the increase of cleaved caspase-3 in Plp-positive mature oligodendrocytes both of *Pten^wt/m3m4^* and *Pten^m3m4/m3m4^*. (B) Western blot of cleaved caspase-3 from cortex lysate of wildtype, heterozygous and homozygous mutant mice. (C) Densitometries of cleaved caspase-3 Western blot. Individual intensity of wildtype samples was set to 1 based on averaging intensity of bands and heterozygous and homozygous mutant values were graphed as percentage of control. Scale Bar = 100 μm. * p < 0.05; Kruskal-Wallis test.


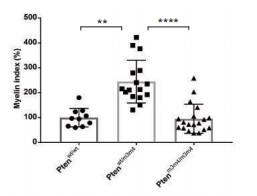


**Supplemental Figure S4.** Myelination index on OL co-culture experiments from Manuscript Figure 5F-K. Quantification of myelin from each group of co-culture revealed increased myelination in DRG neurons co-cultured with *Pten^wt/m3m4^* OLs compared to those of *Pten^m3m4/m3m4^*. ** p <0.01; **** p <0.0001; Kruskal-Wallis test.


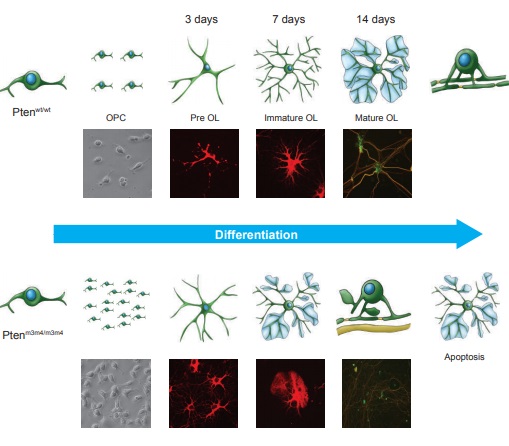


**Supplemental Figure S5.** Schematic representation of the oligodendrocyte lineage development in *Pten^m3m4/m3m4^* mice. OPCs maintain a constant density and uniform distribution throughout the normal brain. As OLs develop, their morphology becomes complex with increased processes. Subsequently, membranous myelinating OLs ensheath axons. However, in the *Pten^m3m4/m3m4^* mouse, OPCs lose their natural program regulating division, migration, differentiation and myelination due to the *Pten^m3m4^* mutation. The mutant shows increased OPC proliferation, migration, and accelerated maturation. Mature OLs of the *Pten^m3m4/m3m4^* genotype show impaired myelin membrane spreading and aberrant myelination. Subsequently, mature OLs, showing disrupted Plp trafficking and ensheathing of axons, undergo apoptosis. Loss of mature OLs might act as a positive signal driving OPC proliferation and maturation. OPC: oligodendrocyte progenitor cell; Pre OL: pre oligodendrocyte; Immature OL: immature oligodendrocyte: Mature OL: mature oligodendrocyte.

**SUPPLEMENTAL REFERENCES**

1. Tilot AK, Gaugler MK, Yu Q, Romigh T, Yu W, Miller RH*,* et al*.* Germline disruption of pten localization causes enhanced sex-dependent social motivation and increased glial production*.* *Hum Mol Genet* 2014; **23**: 3212-3227.
2. McCarthy K, Dde Vellis J. Preparation of separate astroglial and oligodendroglial cell cultures from rat cerebral tissue*.* *J Cell Biol* 1980; **85**: 890-902.
3. Barres BA, Lazar MA, Raff MC. A novel role for thyroid hormone, glucocorticoids and retinoic acid in timing oligodendrocyte development*.* *Development* 1994; **120**: 1097-1108.
4. Noble M, Murray K. Purified astrocytes promote the in vitro division of a bipotential glial progenitor cell*.* *EMBO J* 1984; **3**: 2243-2247.
5. Temple S, Raff MC. Differentiation of a bipotential glial progenitor cell in a single cell microculture*.* *Nature* 1985; **313**: 223-225.
6. Raff MC, Mirsky R, Fields KL, Lisak RP, Dorfman SH, Silberberg DH*,* et al*.* Galactocerebroside is a specific cell-surface antigenic marker for oligodendrocytes in culture*.* *Nature* 1978; **274**: 813-816.
7. Armstrong RC, Harvath L, Dubois-Dalcq ME. Type 1 astrocytes and oligodendrocyte-type 2 astrocyte glial progenitors migrate toward distinct molecules*.* *J Neurosci Res* 1990; **27**: 400-407.
8. Milner R, Anderson HJ, Rippon RF, McKay JS, Franklin RJ, Marchionni MA*,* et al*.* Contrasting effects of mitogenic growth factors on oligodendrocyte precursor cell migration*.* *Glia* 1997; **19**: 85-90.
9. Zuchero JB. Purification and culture of dorsal root ganglion neurons*.* *Cold Spring Harb Protoc* 2014; **2014**: 813-814.

**AUTHORSHIP INFORMATION**

HL, RD, and CE conceptualized and designed the study. HL conducted experiments and contributed to their analysis with assistance from NS. NS helped with targeted experimental design, specifically the IHC experiments and data. HL, ST, RD and CE interpreted the data. HL and ST drafted the manuscript. RD and CE critically revised the manuscript. All authors gave final approval of the manuscript.
